# Comprehensive Statistical Assessment of Post-Thaw Semen Quality in Two Conserved Norwegian Rooster Lines

**DOI:** 10.3390/ani16142181

**Published:** 2026-07-14

**Authors:** Anne Kettunen, Ellen L. F. White, Mervi Honkatukia, Nina Svartedal, Anette Kristine Krogenæs, Jaana Peippo, Amin Sayyari

**Affiliations:** 1 Farm Animals, NordGen—Nordic Genetic Resource Center, Faculty of Biosciences, c/o Norwegian University of Life Sciences, P.O. Box 5003 NMBU, NO-1432 Ås, Norway; ellen-louisa.fagerheim.white@nordgen.org (E.L.F.W.); mervi.honkatukia@nordgen.org (M.H.); 2 Norwegian Genetic Resource Centre, Norwegian Institute of Bioeconomy Research (NIBIO), P.O. Box 115, NO-1431 Ås, Norway; nina.svartedal@nibio.no; 3 Faculty of Veterinary Medicine, Norwegian University of Life Sciences, P.O. Box 5003 NMBU, NO-1432 Ås, Norway; anette.krogenaes@nmbu.no; 4 Natural Resources Institute Finland, Tietotie 4, FI-31600 Jokioinen, Finland; jaana.peippo@luke.fi

**Keywords:** rooster semen, cryobank quality control, flow cytometry, post-thaw holding, poultry genetic resources

## Abstract

The conservation of local poultry genetic resources requires complementary ex situ strategies, particularly when live populations are maintained at a single location. Previous gene bank insemination trials with frozen–thawed semen from Norwegian conserved lines showed acceptable subjective post-thaw motility but unexpectedly low fertilization success, highlighting the need for objective quality-control assessment. The aim of this study was to establish an objective cryobank quality-control baseline for frozen–thawed semen from two Norwegian conserved rooster lines. Specifically, the study evaluated whether routinely applied objective semen-quality assessment methods, including computer-assisted semen analysis (CASA) and flow-cytometric viability assessment, could detect differences between rooster lines and post-thaw handling conditions. Cryopreserved semen doses collected in 2018 and stored in the Norwegian poultry gene bank were thawed and evaluated from 24 roosters representing the Jærhøns and NorBrid8 lines. The samples were originally collected as part of the routine cryopreservation activities of the Norwegian poultry gene bank and therefore did not require specific approval from the Norwegian Food Safety Authority (Mattilsynet). Post-thaw semen quality was assessed using CASA and flow-cytometric viability analysis. The effects of line and short-term refrigerated holding were evaluated using mixed-effects models, supported by minimum detectable effect calculations, correlation analysis, and principal component analysis. Short-term post-thaw refrigerated holding reduced several CASA-derived motility and velocity traits, while viability assessment provided complementary information on membrane integrity. Line-associated differences in selected motility traits and substantial rooster-to-rooster variability in all traits were observed. Multivariate analyses identified major dimensions of covariance among sperm-quality traits without implying direct biological mechanisms of fertility failure. CASA and flow-cytometric viability assessment can provide an objective baseline for cryobank quality control of frozen–thawed rooster semen. However, fertility trials and molecular sperm-integrity markers are required to determine the biological causes of the previously observed discrepancy between motility and fertility.

## Simple Summary

In Norway, the conservation of laying hen lines currently relies entirely on live birds kept in a single gene bank location, creating vulnerability. To protect these genetic resources for the future, Norway needs a long-term cryoconservation strategy whereby semen is safely frozen and stored. The goal of this study was to create a clear starting point for checking the quality of frozen rooster semen stored in the Norwegian poultry gene bank. Frozen–thawed semen from 24 roosters of two Norwegian breeds was examined using computer-assisted sperm analysis and flow cytometry. Short-term refrigerated holding after thawing reduced sperm quality. The two breeds showed differences in selected motility traits, and there were large differences between individual roosters in all traits. These tools provide an objective baseline for checking the quality of frozen semen in the gene bank, but future work should link sperm quality to fertilization success to strengthen Norway’s gene banking efforts.

## 1. Introduction

Local breeds account for most of the world’s poultry genetic diversity [1]. According to The Second Report on The State of the World’s Animal Genetic Resources for Food and Agriculture [2], out of the 1669 chicken breeds worldwide, 1514 (91%) are local, while 155 (9%) are transboundary. However, the competitiveness of commercial breeding companies, limited incentives for local producers, and widespread crossbreeding have accelerated the erosion of local genotypes [3,4]. In 2015, only 212 breeds were classified as “not at risk”, while 1089 had an unknown risk status [2]. Alarmingly, FAO’s follow-up document on status and trends reported a decrease close to 8% in the number of chicken breeds, and no improvement in the percentage of sustainable populations [5]. There are 17 native chicken breeds in the Nordic countries. Of these, 12 are categorized as “endangered” or “vulnerable”, three as “not at risk”, and two have unknown endangerment status [6]. Although numerically few, Nordic landrace breeds represent unique genetic resources whose diversity is increasingly threatened [3]. Developing sustainable conservation strategies is therefore critical to safeguard these populations.

Locally adapted breeds are valuable not only in low-input production systems, but also in high-income countries, where national genetic resources may be at risk because they are often maintained in small, geographically limited populations [1]. Climate change, more frequent outbreaks, and new emerging diseases, as well as recent geopolitical instability, have increased the need for strategic anticipatory preparedness in developed countries, including national and regional planning of food security and non-food self-sufficiency. Local landrace breeds are central to such preparedness because they often harbor unique genetic variation that is absent from commercial lines, particularly for traits likely to be critical for future adaptation. For example, high diversity in the MHC cluster B region of the Finnish Landrace chicken, a genomic feature expected to contribute to disease resistance, has been reported [7]. Similarly, 11 genetic clusters were identified among 13 putatively distinct population units of the Finnish Landrace chicken, which showed high genetic diversity, a clear population structure, and low inbreeding—findings that underscore the conservation value of these populations [8].

Globally, the selective breeding of layer hens and broiler chickens is dominated by a small number of breeding companies. These programs primarily focus on traits that maximize performance under intensive production, e.g., egg number, shell strength, and feed efficiency [1,4]. Their competitive advantage is reinforced by access to substantial resources and advanced biotechnologies, including genomic selection and gene editing. Although consumer pressure for improved animal welfare and organic production has prompted some shifts in breeding objectives, traits related to environmental sustainability and low-input resource use remain largely neglected [1]. Reliance on foreign breeding stock developed for high-input systems therefore undermines efforts to build resilient, self-sufficient food systems in the Nordic region.

Lessons from American cases of culling and genetic erosion, in which valuable poultry lines were irretrievably lost, led to the founding of Norway’s laying hen gene bank in 1973 [9]. Another motivation for creating a nationwide live animal gene bank was the knowledge of rooster semen’s poor suitability for cryopreservation. Jærhøns, the only Norwegian native chicken breed, was among the first to be included in the gene bank; other preserved lines are primarily derived from White Leghorn stock. The gene bank is currently housed at Hvam Upper Secondary School (Nes, Akershus) and is managed in collaboration with the Norwegian Genetic Resource Centre at the Norwegian Institute of Bioeconomy Research (NIBIO), with operational funding from the Ministry of Agriculture and Food.

In 1994, the European Economic Area (EEA) agreement opened the Norwegian market to live genetic material from international commercial companies. As a result, Norwegian breeding material was outcompeted and the national breeding program discontinued [3,9]. Throughout its existence, the gene bank has supplied hatching eggs, chicks, and live birds to hobbyists nationwide. The threats posed by climate change and disease outbreaks, together with inadequate contingency measures to protect local populations, underscore the need for a complementary ex situ cryopreservation strategy for laying hens.

To complement live-animal safeguarding efforts and further secure the breeding lines at Hvam Agricultural College, the Norwegian Genetic Resource Centre conducted two semen collection and cryopreservation trials in 2016 and 2018. These trials yielded a total of 540 cryopreserved semen doses from two poultry lines, Jærhøns and NorBrid8. Based on the trial records, post-thaw sperm motility assessed subjectively by microscopy was relatively high (≤40%), yet the fertilization rates following artificial insemination with thawed semen were extremely low (<4%). The cause of this discrepancy—apparently good post-thaw motility but very poor fertilization—remains unclear and prompted further investigation into post-thaw sperm quality.

This pilot study was motivated by these results, where frozen–thawed semen showed acceptable subjective post-thaw motility but unexpectedly low fertilization success, highlighting the need for objective quality-control assessment. The aim of this study was to establish an objective cryobank quality-control baseline for frozen–thawed semen from two Norwegian conserved rooster lines, rather than to identify the biological causes of reduced fertility. Specifically, the study evaluated whether routinely applied objective semen-quality assessment methods, including computer-assisted sperm analysis (CASA) and flow-cytometric viability assessment, could detect differences between rooster lines and post-thaw handling conditions. In addition, a suite of statistical methods was used exploratorily to characterize patterns among CASA-derived sperm-quality traits within a cryobank quality-control context. This integrative framework aimed to provide a clearer and more objective basis for interpreting sperm performance and to guide decisions about future work, including experimental design and the choice of assessment methods. Such future work will help improve donor selection and strengthen ex situ cryobanking efforts for Norwegian laying hen lines [10].

## 2. Materials and Methods

### 2.1. Animal Material and Collection of Semen

The Norwegian gene bank for laying hens maintains 12 different breeds or breeding lines. Birds are housed in specially designed enriched cages, each equipped with perches, a sand bath, and a nest box. The cages have increased height to allow natural behavior and successful mating. Each cage contains one rooster and five to seven hens, forming a breeding family. For each breed, 20–30 families are maintained at any given time within a rotational breeding system, in which roosters are systematically rotated among families to restrict the rate of inbreeding to ≤0.5% per generation.

**Figure 1:**
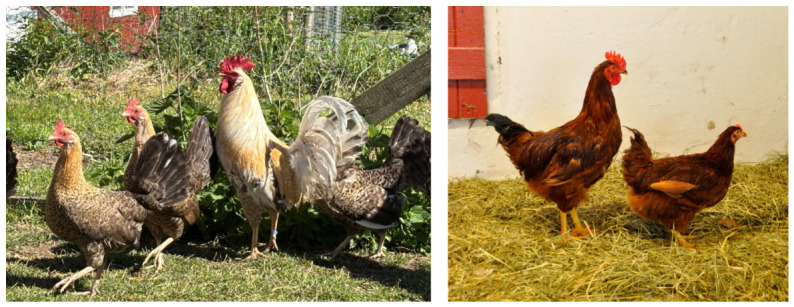
Jærhøns (**left**) and NordBrid8 (**right**) individuals from Hvam live animal gene bank.

Semen was collected in 2018 from two genetic backgrounds, the Jærhøns (JH) breed and the NorBrid8 (NB8) breeding line, as part of the Norwegian poultry gene bank cryopreservation activities (Figure 1). From hatching until 16 weeks of age, chicks were reared in large, breed-specific groups of approximately 120 individuals. At 16 weeks post-hatch, 72 roosters (35 JH, 37 NB8) from the 2018 reproduction cohort, and not included in the routine gene-bank breeding activities, were selected for semen collection and moved into enriched cages, with six males per cage. The poultry house was equipped with an automatic chain-feeding system operating three times daily, and at 35 weeks of age, the light regime was set to 16 h of light per day. Three weeks prior to the start of the project, at 32 weeks of age, semen was collected from the roosters twice weekly to habituate them to the procedure. During the experimental period, semen was subsequently collected every second day. On average, 2.49 and 1.76 collections were performed for JH and NB8, respectively.

The semen collection technique used in the 2018 trial was the standard method applied in the Norwegian gene bank before 2000, when hens were routinely inseminated with fresh semen (personal communication, Nina Svartedal). Sperm processing prior to freezing followed the protocol provided by the Centre for Genetic Resources, the Netherlands (CGN, Wageningen University & Research). In short, a modified classical chicken diluent (Lake’s 7.1. diluent) and dimethylacetamide (DMA) were used for sample preparation prior to freezing. Straw preparation and freezing followed the procedures described in the CGN working instruction WRK-AGR-100 (copyrighted protocol).

### 2.2. Experimental Design and Sperm Quality Assessment

**Figure 2:**
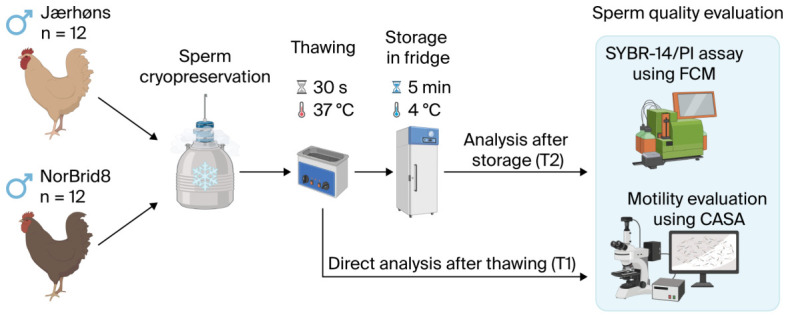
Experimental design for assessment of sperm quality in roosters from two breeds in the Hvam gene bank using computer-assisted sperm analysis (CASA) and flow cytometry (FCM).

Cryopreserved semen doses collected in 2018 and stored in the Norwegian poultry gene bank were thawed and evaluated in this study. Semen quality was evaluated using computer-assisted sperm analysis (CASA) and flow cytometry on 24 doses originating from 12 JH and 12 NB8 roosters. Each dose corresponded to one rooster (single ejaculate) and was evaluated under two post-thaw holding conditions, T1 and T2, providing paired observations per rooster (Figure 2).

Motility and kinematic traits were quantified using a CASA system (Sperm Class Analyzer, Microptic S.L., Barcelona, Spain) connected to a Nikon phase-contrast microscope (Nikon corporation, Tokyo, Japan) equipped with a 10× objective and a 37 °C stage warmer. CASA settings (velocity thresholds, track length, and cell size detection limits) were optimized for rooster spermatozoa based on preliminary tests and were kept constant across analyses. The CASA settings used were as follows: frame rate, 25 frames/s for 25 consecutive frames (one second recording period); particle detection area, 5–190 µm^2^. For each sample, at least three non-overlapping fields were recorded, yielding ≥200 analyzed spermatozoa. Each sample underwent two CASA assessments, referred to as treatments in this study. In the first treatment (T1), motility and kinematics were evaluated immediately after thawing the straws in a 37 °C water bath for 30 s, whereas for the second treatment (T2), the remaining portion of the sample was stored at 4 °C for up to 5 min before analysis to evaluate sensitivity to short-term refrigerated holding. This assessment was included to reflect practical laboratory evaluation or insemination scenarios, where brief cooling periods may be unavoidable. Accordingly, it served to evaluate the short-term resilience of thawed semen within a cryobank quality-control context rather than to propose an optimal holding protocol. Because T1 and T2 were sequential post-thaw handling points, their order was not randomized.

After thawing, semen samples were diluted in pre-warmed 0.9% NaCl solution to a final concentration of approximately 50 × 10^6^ sperm/mL and loaded into a pre-warmed disposable chamber with a depth of 20 µm (Leja, B.V., Nieuw-Vennep, The Netherlands). The following parameters were recorded: total motility (MOT, %), progressive motility (PROG, %), curvilinear velocity (VCL, µm/s), average path velocity (VAP, µm/s), straight-line velocity (VSL, µm/s), straightness (STR = (VSL/VAP) × 100, %), and linearity (LIN = (VSL/VCL) × 100, %). Total motile spermatozoa were defined as VCL > 15 µm/s, and progressive spermatozoa as VCL > 30 µm/s with STR > 70%.

Sperm viability was assessed using flow cytometry using the LIVE/DEAD™ Sperm Viability Kit (L7011; Invitrogen™, Thermo Fisher Scientific, Eugene, OR, USA), following the manufacturer’s instructions. For this assay, one thawed sample per rooster (T1) was diluted in PBS to 1–5 × 10^6^ sperm/mL, stained with SYBR-14, and incubated for 10 min at 37 °C in a sealed cabin in the dark, followed by the addition of propidium iodide (PI) and an additional 5 min incubation. Samples were analyzed using a CytoFLEX S flow cytometer (Beckman Coulter, Inc., Brea, CA, USA), with fluorescence signals detected in the FL1 channel for live and FL3 channel for dead spermatozoa. A total of 10,000 events were recorded per sample. Live spermatozoa were defined by positive SYBR-14 (green) staining in the absence of PI (red) fluorescence. Accordingly, only SYBR-14-positive/PI-negative events were classified as viable spermatozoa, while all PI-positive events, including double-positive spermatozoa, were considered membrane-compromised and nonviable. The viability percentage (VIA, %) was calculated as the proportion of live spermatozoa over the sum of live, dead, and nonviable spermatozoa.

### 2.3. Statistical Methods

#### 2.3.1. Mixed Models

To evaluate the effects of breed and treatment on CASA-derived sperm motility and kinematic traits, linear mixed-effects models with breed and treatment as fixed effects, and rooster as a random intercept to account for repeated measurements were fitted. Each rooster contributed one ejaculate assessed under both treatments (T1 and T2), making treatment a within-subject factor and breed a between-subject factor. Because each ejaculate was divided into two treatment subsamples, “rooster” was included as a random effect to model the paired structure of the data rather than repeated ejaculates. The final model was specified as follows:

(1) $$y_{ijk}=\mu +{breed}_{i}+{treatment}_{j}+u_{k}+\varepsilon_{ijk},$$

where $y_{ijk}$ is the observed value of the sperm motility/kinematic trait for the *k*-th rooster of breed *i* under treatment *j*, μ  is the overall mean, *breed_i_* and *treatment_j_* are fixed effects, $u_{k}\sim \left(0,\sigma_{u}^{2}\right)$ is the random intercept for the rooster, and $\varepsilon_{ijk}\sim \left(0,\sigma^{2}\right)$ is the residual error. Models were fitted in R 4.5.1 [11] using the lme4 package [12]. Type III ANOVA and *p*-values for fixed effects were obtained using the lmerTest package [13]. For each trait, an initial mixed model including the breed-by-treatment interaction was fitted. The significance of the interaction was evaluated using a likelihood-ratio test (α = 0.05) by comparing the full model with a reduced model lacking the interaction. As no breed-by-treatment interactions were detected for any trait, the effects of breed and treatment were treated as additive in the final models.

The flow cytometry viability trait (VIA) was analyzed using one-way ANOVA model of the following form:

(2) $$y_{ij}=\mu +{breed}_{i}+\varepsilon_{ij},$$

where $y_{ij}$ is the observed value of the sperm viability trait (VIA) for the *j*-th rooster of breed *i*, μ  is the overall mean, *breed_i_* is the fixed effect of breed, and $\varepsilon_{ij}\sim \left(0,\sigma^{2}\right)$ is the residual error. The homogeneity of variances was tested using Levene’s test implemented in the car package [14].

#### 2.3.2. Principal Component Analyses

Because the CASA-derived motility and kinematic traits are biologically interdependent and statistically correlated, the univariate mixed-effects models were complemented with principal component analysis (PCA) to characterize the multivariate structure of sperm performance. PCA was conducted on the seven CASA traits (MOT, PROG, VCL, VAP, VSL, STR, LIN) using the prcomp function in R, with variables centered and scaled to unit variance. The analysis yielded eigenvalues, loadings, and the proportion of variance explained for each component, with absolute loadings ≥ 0.35 considered indicative of a substantial contribution.

The PC scores for the first three components (PC1–PC3) were appended to the dataset and subsequently analyzed using the same linear mixed-effects framework applied to the original CASA traits, with breed and treatment as fixed effects and rooster as a random intercept. PCA biplots were generated using the factoextra package [15], displaying individual scores in the PC1–PC2 space together with variable loadings and 95% normal ellipses for breed and treatment. This multivariate approach provided an integrated summary of motility and kinematic patterns and allowed us to assess whether breeds and treatments differed not only in individual traits but also in the underlying covariance structure of sperm movement. Within-rooster changes across treatments were visualized using spaghetti plots, where each male’s direct-thawed and refrigerated samples were connected in PCA space to illustrate individual trajectories.

#### 2.3.3. Correlation Analysis

To characterize the pairwise relationships among CASA-derived motility and kinematic traits, Pearson’s correlation coefficients were computed separately for each breed and treatment. Correlation matrices were generated using complete-case observations, and significance levels were obtained using standard parametric tests. Because the CASA traits are interdependent, these analyses provided a complementary perspective to the mixed-effects models by revealing the degree to which motility traits covary within each breed and within each treatment. In addition to the CASA traits, correlations between viability and motility and kinematic variables were examined to assess whether sperm viability aligns with specific aspects of sperm movement. Correlation matrices were visualized as heatmaps, and pairwise scatterplots with fitted regression lines were produced to illustrate the strength and direction of associations. Pairwise plots were generated using the GGally package [16]. Additional visualization was performed using ggplot2 [17]. All correlation analyses were performed in R using functions from the base stats package.

### 2.4. Sensitivity Analysis Through Power Calculations and Minimum Detectable Effects

In addition to the primary statistical tests, retrospective statistical power analyses were conducted, and minimum detectable effects (MDEs) were estimated to contextualize both CASA and flow cytometry traits. Because this was a pilot study, no a priori power calculations were performed. The statistical power in small experiments is highly sensitive to assumptions about variance structure and model specification; therefore, simulation-based approaches were used to evaluate the sensitivity of our design. The retrospective power and MDE analyses were intended to contextualize the sensitivity of the study and to guide future experimental design, rather than to compensate for the modest sample size. Their purpose was to highlight where the study lacked power to detect effects of biologically meaningful magnitude. Accordingly, findings for traits with low power or with confidence intervals (CIs) overlapping the MDE threshold should be interpreted cautiously and considered provisional until validated in larger studies.

Power analyses were performed using simulation models implemented in the Superpower package (Monte Carlo simulation) in R [18], incorporating empirical group means and pooled standard deviations for each trait. For the CASA variables, simulations followed a mixed-design structure with breed treated as a between-rooster factor and treatment as a within-rooster factor, using the observed correlations between paired measurements to account for within-individual dependence. These simulations generated trait-specific estimates of power for detecting breed and treatment effects. Interaction effects were not included in the power summary because they were not significant in the final mixed-model analysis.

For VIA, power was simulated using a two-level between-subjects design with breed as the sole fixed factor, applying the same simulation framework but without a component accounting for repeated measurements. This ensured that the power estimates for these traits reflected the appropriate experimental structure while remaining directly comparable to the CASA-based simulations.

In addition to reporting *p*-values and simulated power estimates, minimum detectable effects (MDEs) were calculated to evaluate the sensitivity of the study design. The MDE represents the smallest effect size that could be reliably detected with a predefined power level—here, 80% at α = 0.05. MDEs were derived analytically from the observed standard errors of the breed and treatment effects, using a closed-form power approximation implemented in power.*t*.test. These analytical MDEs quantify the minimum raw effect size—and corresponding standardized effect size—that the study was sufficiently powered to detect given the actual sample size and variance structure. By comparing the 95% CIs of the estimated effects with the corresponding MDE thresholds, it was assessed whether the study could detect effects of practical relevance. This complements significance testing by shifting the interpretive focus from whether an effect is statistically significant to whether it is large enough to be biologically meaningful.

## 3. Results

### 3.1. Sperm Motility and Viability Across Breeds, Treatments, and Roosters

Across all samples, frozen–thawed semen showed an average motility of 36.46% and a progressive motility of 19.94%. The mixed-effects models revealed a clear effect of breed on MOT and PROG (Table 1), where NB8 roosters exhibited lower MOT (–7.85 percentage points) and PROG (–5.10 percentage points) than JH. No significant breed effects were detected for any of the kinematic traits.

Short-term post-thaw refrigerated holding (T2) reduced several traits, with decreases of –6.67 percentage points for MOT, –3.92 percentage points for PROG, –3.77 µm/s for VCL, –4.27 µm/s for VAP, –4.09 µm/s for VSL, and –1.77 percentage points for LIN. STR showed no significant treatment effect.

For the flow cytometry traits, JH roosters had 5.43 higher VIA than NB8; this difference was not statistically significant (Table 1).

**Figure 3:**
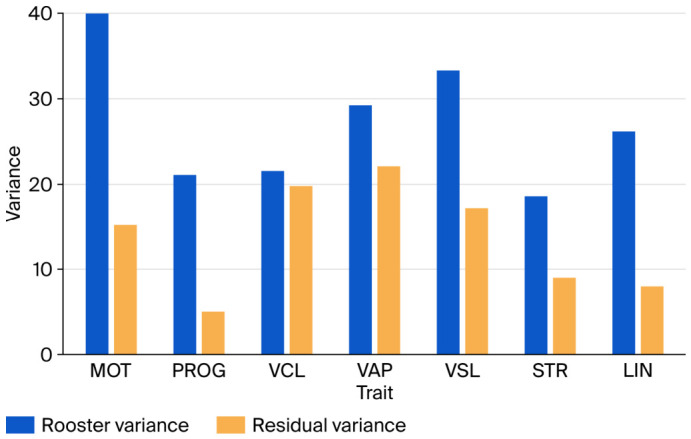
Variance components across CASA traits from the mixed-effects model analysis.

Mixed-model analyses revealed clear rooster-specific differences across all CASA traits (Figure 3). For motility traits, roosters accounted for a substantial proportion of the total variance, with rooster variance exceeding residual variance for both MOT (39.86 vs. 15.13) and PROG (21.04 vs. 4.99), indicating highly repeatable performance within roosters. A similar pattern was observed for all kinematic traits; rooster variance was consistently larger than the residual component, where only VAP showed nearly equal rooster and residual variance (29.17 vs. 22.12). The significant random-effect variance reflects differences among roosters in the baseline quality of the single ejaculate each provided, rather than repeatability across ejaculates or stable rooster-specific effects across time.

### 3.2. Sensitivity Analyses

**Figure 4:**
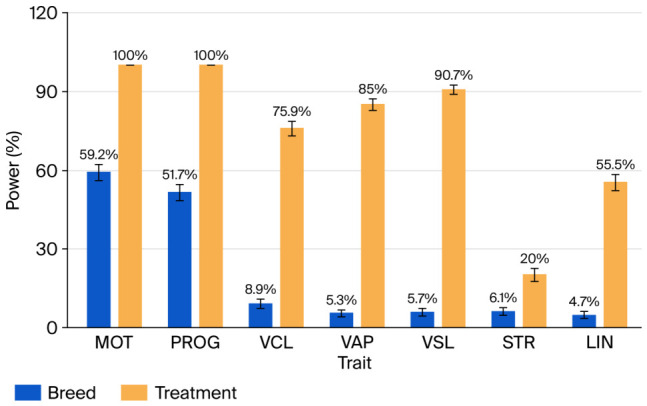
Simulated trait-specific statistical power estimates for breed and treatment effects for CASA traits.

To assess the robustness of these findings, sensitivity analyses based on simulation-based power estimates and minimum detectable effects were conducted. Using thresholds > 0.80 for high power, 0.50–0.80 for moderate power, and <0.50 for low power, the simulations showed that the study had high power to detect treatment effects for MOT, PROG, VAP, and VSL, whereas power was moderate for VCL and LIN, and low for STR (Figure 4). Breed effects showed moderate power for MOT and PROG, and very low power for VCL, VAP, VSL, STR, and LIN, with none of the breed-level estimates reaching the 0.80 threshold for a high-powered experiment. Flow cytometry-based VIA, which was measured once per rooster and analyzed using a between-subject design, showed low power below 0.35.

**Figure 5:**
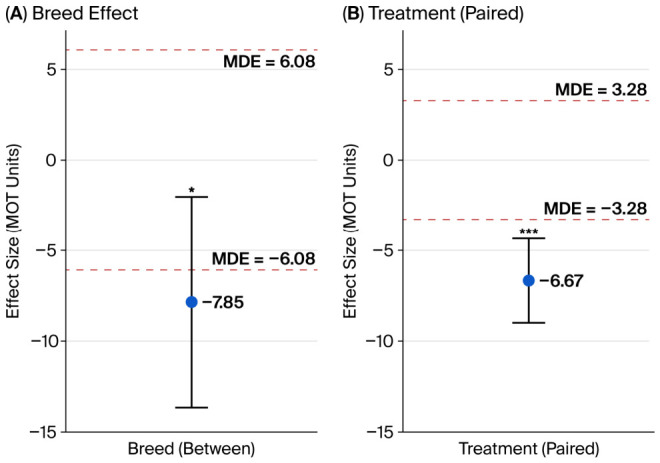
Observed effect estimates with ±95% confidence intervals (CIs) for the breed (**A**) and treatment (**B**) effects compared with the minimum detectable effect (MDE) thresholds for MOT. Dashed lines indicate positive and negative MDE values, with bold labels showing their magnitudes. Numerical point estimates and significance indicators are shown above each effect; * and *** indicate statistical significance at *p* < 0.05 and *p* < 0.001, respectively.

**Figure 6:**
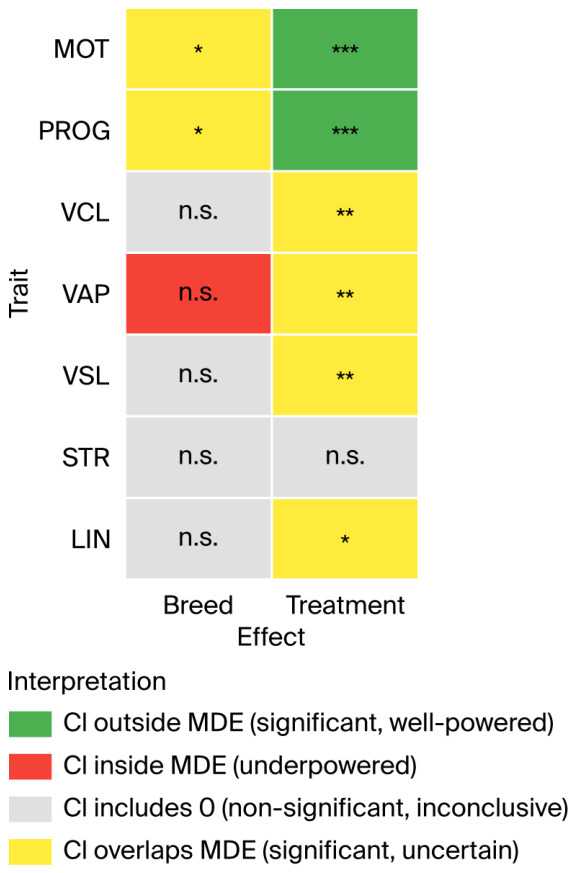
Traffic light interpretation combining linear mixed-model significance and CI vs. MDE band for CASA traits; *, **, *** indicate statistical significance at *p* < 0.05, *p* < 0.01, and *p* < 0.001, respectively.

To evaluate whether the study could detect effects of practical relevance, observed CIs with the corresponding MDE thresholds were compared. Each effect was classified according to the relationship between its CI and the MDE band as (i) significant and well-powered when the CI lay entirely outside the MDE band, (ii) significant but uncertain when the CI overlapped with the MDE band, (iii) underpowered when the CI fell completely with MDE band, and (iv) non-significant-inconclusive when the CI included zero but extended outside MDE band. Figure 5 illustrates this classification for MOT, where the breed effect (Figure 5A) was significant but uncertain (category ii), whereas the treatment effect (Figure 5B) was significant and well-powered (category i). Applying this classification across traits showed that only the treatment effect for MOT and PROG was both statistically significant and well-powered, indicating a conclusive unfavorable impact of refrigeration on these traits (Figure 6). In contrast, the breed effect for MOT and PROG, as well as the treatment effects for VCL, VAP, VSL, and LIN, fell into category ii. The CIs for the breed effects for VCL, VSL, STR, and LIN, and treatment effect CI for STR barely exceeded the MDE band and were close to being classified as category iii, and as “red” in Figure 6. 

For the flow cytometry trait, the breed effects for VIA were non-significant and inconclusive (category iv). Trait-specific MDE plots for all the CASA and flow cytometry traits are provided in Figure S1.

### 3.3. Correlations

**Figure 7:**
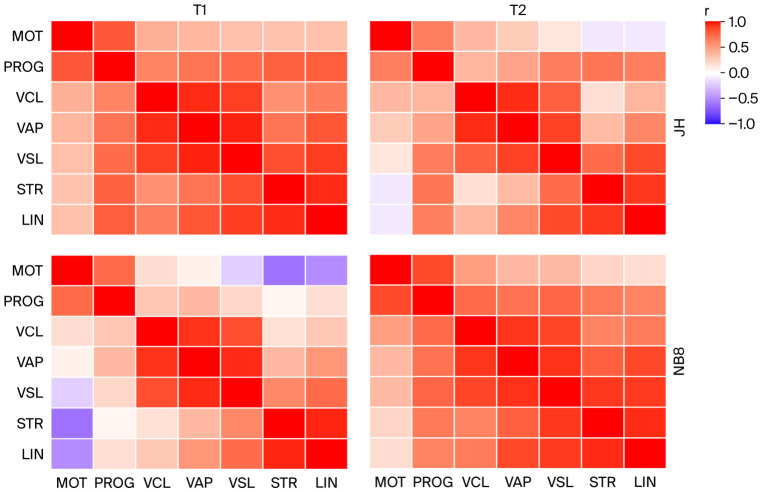
Pearson’s correlation (r) matrices for CASA-derived motility and kinematic traits in JH and NB8 roosters under the two treatments (T1 and T2).

Pairwise correlations among sperm motility and kinematic traits were examined to explore potential covariation within each breed and treatment. Given the small sample size, these analyses should be considered exploratory. Nonetheless, some breed-specific patterns were apparent (Figure 7). The correlations between MOT and kinematic parameters were inconsistent and non-significant in both breeds across treatments, with the exception of a significant negative correlation between MOT and STR in JH8 in T1 (−0.61). JH roosters displayed relatively strong correlations (0.62–0.79) between PROG and all kinematic traits in both treatments, except for the non-significant PROG-VCL and PROG-VAP correlation in T2. In contrast, the PROG-kinematics correlations in NB8 were low and non-significant in T1, but high and significant (0.62–0.76) in T2. In general, the correlations between kinematic parameters were high and significant in both JH and NB8 across treatments. This said, the VCL/VAP-STR/LIN correlations were low and non-significant for NB8 in T1 but shifted to significant in T2. A partly opposite pattern was displayed by JH, where the correlation between VCL and LIN and VAP and STR shifted from significant to non-significant between T1 and T2. Notably, the weak positive or negative associations between MOT and kinematic parameters in NB8 shifted to positive values in T2 (Figure 7). Full correlation matrices for all traits and groups are provided in Figure S2.

**Figure 8:**
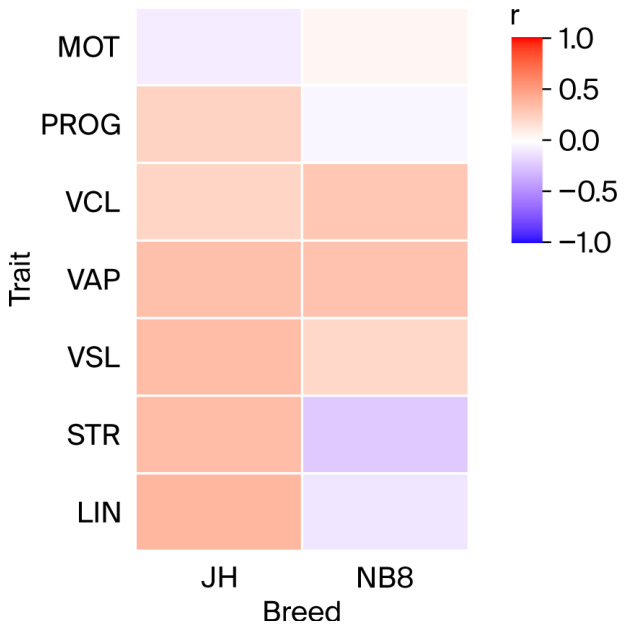
Pearson’s correlation (r) matrix for flow cytometry-derived viability percentage and CASA-derived motility and kinematic traits in JH and NB8 roosters.

The correlations between VIA and CASA traits are displayed in Figure 8. In both JH and NB8, the correlations between MOT and VIA were uniformly close to zero. In JH, all other correlations between VIA and kinematic traits were low and positive, whereas NB8 showed low negative tendencies with STR and LIN. None of these correlations were statistically significant.

### 3.4. PCA

PCA of the CASA variables identified several statistically distinct axes of variation. The interpretation focused on variables with absolute loadings ≥ 0.35, which were considered to represent moderate to strong contributions to each component (Table 2). PC1 showed strong positive loadings for all velocity- and linearity-related traits (PROG, VCL, VAP, VSL, STR, and LIN), representing a general axis of overall sperm movement quality. PC2 displayed opposing loadings for motility traits (MOT, PROG; negative loadings) versus straightness and linearity (STR, LIN; positive loadings). This component therefore reflects a statistical contrast between motility and linearity and should not be interpreted as direct evidence of a biological trade-off. PC3 differentiated curvilinear velocity (VCL, VAP; positive) from linearity and progressive movement (STR, PROG; negative), capturing variation in movement pattern. Together, PC1–PC3 explained 98.28% of the total variance in CASA traits (PC1: 65.69%, PC2: 20.80%, and PC3: 11.79%), and interpretation was therefore restricted to these components (Table 2).

Mixed-effects models showed that treatment had a strong effect on PC1, indicating that short-term refrigerated holding substantially influenced overall sperm movement quality (Table 3). Breed had no effect on PC1. For PC2, both breed and treatment had significant effects, consistent with differences between breeds in the motility–linearity contrast and with treatment-induced shifts in this axis. PC3 showed no significant effects of either factor.

The effect-size estimates were consistent with mixed-model results (Table 3). For PC1, treatment had a large effect (η^2^_p_ = 0.44), whereas the effect of breed was very small (η^2^_p_ = 0.02), confirming that short-term refrigerated holding strongly influenced overall sperm movement quality while breed differences were negligible. For PC2, both breed (η^2^_p_ = 0.23) and treatment (η^2^_p_ = 0.37) showed moderate to large effects, indicating that both genetic background and treatment contributed to variation in the motility–linearity contrast. The effects on PC3 were small for both breed and treatment (η^2^_p_ ≤ 0.11). Marginal R^2^ values indicated that fixed effects explained 10.48%, 24.40%, and 6.88% of the variance in PC1–PC3, respectively, while conditional R^2^ values (35.63–77.92%) highlighted substantial rooster-to-rooster variation across all components.

**Figure 9:**
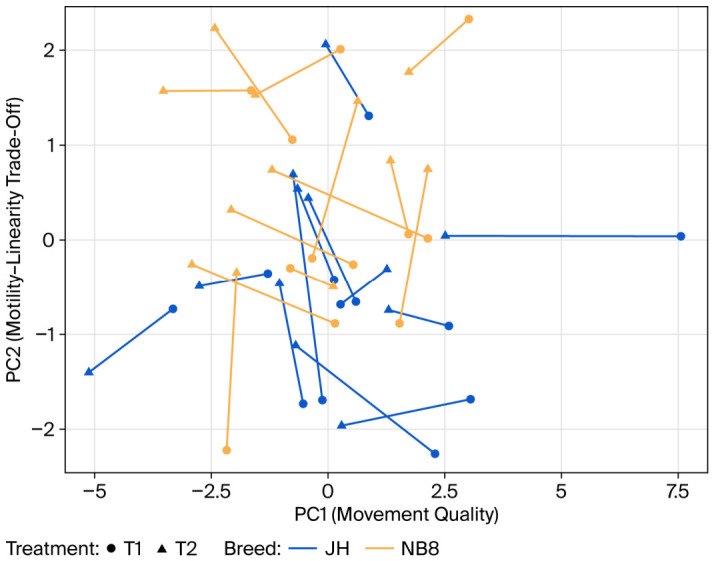
Spaghetti plots showing within-rooster trajectories across treatments in PCA space, illustrating individual-level change along PC1 (overall movement quality) and PC2 (contrast between motility-related and linearity-related traits).

Although the mixed models indicated no breed differences in mean PC1 scores, the spaghetti plots revealed clear differences in within-breed variability (Figure 9). NB8 roosters showed tightly clustered and relatively short trajectories along PC1, indicating consistent movement quality and a uniform response to refrigeration. In contrast, JH roosters exhibited larger and more variable shifts along PC1, demonstrating greater individual heterogeneity in both baseline sperm quality and treatment sensitivity. These variance patterns are not captured by the fixed-effect tests but are consistent with the high conditional R^2^ values, which indicate substantial rooster-to-rooster variation across components.

**Figure 10:**
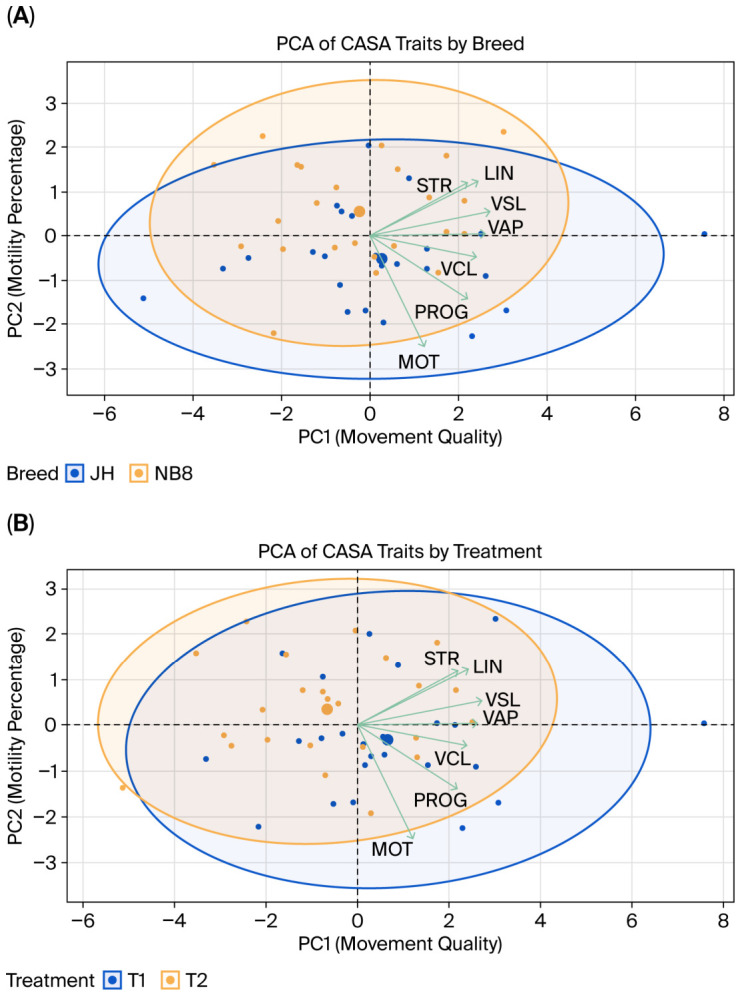
PCA of CASA traits displayed separately for (**A**) breed and (**B**) treatment. Individual roosters are projected into the PC1–PC2 space, with 95% normal ellipses indicating group dispersion.

In the PCA biplots, treatment groups showed a clear shift along PC1, consistent with the strong treatment effect on overall movement quality (Figure 10). However, the treatment ellipses overlapped substantially, reflecting high within-treatment variability. In contrast, breed differences were most evident along PC2, which captures a covariance pattern contrasting motility-related and linearity-related traits. Because the within-breed variation along this axis was lower, breeds showed a clearer visual separation in the PCA space.

## 4. Discussion

Following decades of use in genetic conservation programs, cryopreservation remains an essential tool for safeguarding animal genetic resources and supporting breeding strategies [4,19,20,21,22]. However, while frozen–thawed semen performs well in many mammalian species, fertilization success in poultry—especially roosters—remains inconsistent and generally low [1,19,23]. The present study was therefore designed as a cryobank quality-control investigation rather than a mechanistic fertility study. It was motivated by previous gene-bank insemination trials in which frozen–thawed semen displayed acceptable subjective post-thaw motility yet resulted in unexpectedly low fertilization rates. Rather than attempting to identify the biological causes underlying this discrepancy, the objective was to assess whether objective analytical methods routinely used in semen quality control could detect differences in post-thaw semen quality among genetic lines and under short-term post-thaw refrigerated holding conditions.

Objective semen quality assessments, CASA and flow cytometry, provide standardized and reproducible indicators of post-thaw motility, kinematic performance, and membrane integrity, and are widely regarded as primary benchmarks of cryopreserved semen quality. However, increasing evidence shows that cryopreservation-induced damage extends beyond these conventional parameters. In poultry, cryoinjury involves multiple cellular and molecular pathways, including membrane destabilization, oxidative stress, mitochondrial dysfunction, and DNA damage [24]. Recent work further indicates that post-thaw handling procedures can influence not only motility and viability but also DNA integrity, underscoring the multifactorial nature of cryopreservation outcomes [25]. Evidence from other production animals similarly highlights sperm DNA integrity as a potentially important component of male fertility assessment, although its predictive value remains species-dependent and incompletely resolved [26,27]. Because the present study aimed to establish an objective baseline for post-thaw quality of archived cryobank material rather than to investigate mechanisms of fertility variation, molecular endpoints such as DNA fragmentation, chromatin integrity, or oxidative stress markers were not included. Nonetheless, integrating these measures—together with fertilization trials—represents an essential next step toward a more comprehensive understanding of reproductive performance following cryopreservation.

The distinctive morphology and physiology of poultry sperm contribute substantially to their cryo-sensitivity. Rooster sperm are filiform, with a narrow head and long tail, making them particularly vulnerable to mechanical and osmotic stress during freezing and thawing [4,19]. Their plasma membranes contain high proportions of polyunsaturated fatty acids and relatively little cholesterol, resulting in elevated membrane fluidity [20,21]. This increased membrane fluidity increases their permeability to ions and small molecules but also heightens the risk of leakage and destabilization under cryo-stress. Combined with limited antioxidant defenses, these features predispose rooster sperm to loss of motility and viability, membrane rupture, DNA fragmentation, and reduced fertilization capacity [19,20,21]. Compounding these challenges, many cryoprotectants (CPAs) that are effective in mammals are either toxic or contraceptive in poultry [19]. Consequently, no universally effective, scalable protocol for rooster semen cryopreservation has yet been established.

Beyond intrinsic biological sensitivity, several practical factors influence the success of frozen–thawed poultry semen. Environmental stressors such as excessive light, temperature fluctuations, and handling stress can reduce sperm viability [23]. Gentle collection techniques and strict hygiene protocols are essential to minimize mechanical damage and contamination.

In the context of the Norwegian gene bank, additional management-related factors may also contribute to semen quality. The gene bank is located at an upper secondary school, and students conduct practical training in the henhouse three days per week. This regular human presence means that the birds are more accustomed to people and generally calmer than flocks kept in commercial facilities. Students participate in daily husbandry tasks and routinely handle some of the birds, which likely increases the birds’ tolerance to handling and reduces stress during semen collection procedures.

The post-thaw motility traits in the present study were moderate, with an average total motility of approximately 36% and progressive motility around 20%. These values fall at the lower end of the post-thaw motility range commonly reported for rooster semen evaluated by CASA, which typically spans 30–65% depending on the thawing protocol, extender composition, CPAs, antioxidant supplementation, storage duration, and CASA settings [20,28,29]. Studies using DMA, for example, have reported total motility between 44.4% and 60.5% and progressive motility between 23.5% and 33.8%, influenced by the sperm concentration and DMA levels [30]. Comparisons with earlier work further highlight that most published motility and kinematic values exceed those observed here for JH and NB8. Ehling et al. reported uniformly high motility and kinematic traits in pooled ejaculates from three Leghorn lines, with VCL and VSL nearly double those in our samples [31], and their later study on individual ejaculates [32] documented even higher total (41.9–47.3%) and progressive motility (27.4–34.9%) alongside consistently elevated kinematic parameters. Long et al. observed kinematic ranges across eight pedigreed layer lines that broadly overlapped with our findings, though their VCL values were higher and LIN values lower [33]. In contrast, Madeddu et al. reported total motility slightly below that of our samples but extremely low progressive motility (2.9–3.5%) and generally reduced kinematic values, particularly for VCL, VAP, and VSL [34]. Taken together, the motility values in the present study align with the lower to mid-range of published post-thaw outcomes, while the kinematic traits—although within the broad variability reported across breeds and protocols—tend to be lower than those documented in studies using optimized cryopreservation conditions or genetically distinct lines.

Mixed-model analyses were used to evaluate the effects of breed and short-term post-thaw refrigerated holding on motility, kinematic traits, and viability. Short-term refrigerated holding had a significant adverse effect on all CASA traits, except STR. Although storing rooster semen at 4 °C after thawing may appear counterintuitive, this assessment was included to evaluate short-term resilience rather than to propose an optimal handling protocol. Thawing at 37 °C followed by refrigerated post-thaw holding may impose additional cooling stress on spermatozoa and influence motility, viability, metabolism, and structural integrity [29], although post-thaw cooling effects are known to be species- and protocol-dependent. While one study reported that samples thawed at 37 °C and stored at 4 °C could retain sperm quality for a limited period [20], our results showed a more rapid decline in sperm traits after short-term refrigerated holding, indicating that the cryobank samples used here were more vulnerable to secondary cooling and rewarming stress. Thus, the second post-thaw assessment (T2) should be interpreted as a practical test of sperm stability under short-term refrigerated holding and rewarming, rather than a recommendation for routine semen handling.

Significant breed differences were detected only for MOT and PROG, with JH roosters exhibiting higher total and progressive motility than NB8, while no breed effects were observed for any kinematic parameters. These results are partly consistent with earlier findings. Ehling et al. detected line differences only in VCL and VAP among three conservation lines [32], whereas Long et al. reported broader differences across eight pedigreed layer lines, including VCL, VAP, VSL, STR, and LIN [33]. Interestingly, Madeddu et al. reported significant breed differences in CASA traits for fresh semen, whereas breeds did not differ when CASA was performed on frozen–thawed semen [34]. Similarly, Li et al. reported significant breed effects across all CASA traits in fresh semen from seven breeds [35]. Taken together, these studies indicate that breed-specific variation in CASA traits is evident, but the magnitude and direction of these differences vary across traits and experimental contexts.

The mixed-model results revealed rooster-specific differences in CASA traits. As both treatments were applied to the same ejaculate, the random intercept structure captured baseline quality differences among roosters rather than repeatability across ejaculates or temporal stability of semen quality. Several traits—particularly MOT, PROG, VSL, STR, and LIN—showed substantially greater between-rooster variance than residual variance, indicating that these aspects of sperm performance were highly consistent within roosters across treatments. Traits with more balanced variance components, such as VCL and VAP, nevertheless still exhibited clear rooster-level differences. The multivariate analyses supported this pattern. Large conditional R^2^ values (0.36–0.78) demonstrated that rooster identity accounted for a large proportion of the total phenotypic variation, whereas fixed effects contributed more modestly. Spaghetti plots further illustrated this pattern by showing that roosters differed not only in their average sperm performance but also in the stability of their responses across treatments, with some males exhibiting consistent trajectories while others showed markedly greater variability.

The substantial between-rooster variance observed here should not be interpreted as evidence of high additive genetic variance. Instead, individual differences in CASA traits may arise from permanent environmental effects, ejaculate-specific variation, non-additive genetic factors, or the expression of different recessive alleles across individuals. Although heritability estimates for avian sperm quality traits are limited and largely based on fresh semen, existing studies suggest that several CASA traits have a genetic component. Published estimates for motility and progressive motility vary widely across breeds and study designs. Bongalhardo et al. reported low to moderate heritability for motility (0.08–0.36 ± 0.16–0.18) in White Leghorn [36], whereas Kabir et al. estimated a much higher heritability for progressive motility (0.83 ± 0.04) in a crossbred line [37]. Hu et al. reported high heritability for viability (0.52) and motility (0.85) in Beijing-You chickens, along with positive genetic and phenotypic correlations between motility and viability [38]. In contrast to these correlations, essentially zero correlations between VIA and MOT were observed in both breeds in this study. Importantly, the conservation lines used here are maintained under a rotational breeding scheme involving 20–30 families per generation, which minimizes the formation of sub-clusters despite high inbreeding. Taken together, these findings indicate that while genetic factors may contribute to variation in sperm performance, the strong rooster-level differences detected here likely reflect a combination of physiological, environmental, and ejaculate-specific influences rather than heritable additive effects alone.

Because mixed-model analyses revealed several breed and treatment differences in CASA traits, an evaluation was conducted to determine whether the study had sufficient sensitivity to detect effects of biologically meaningful size. First, trait-specific retrospective power simulations were conducted, followed by MDE calculations, to contextualize the robustness of the observed effects. These analyses highlight that the statistical power varies substantially across traits in small-scale studies due to differences in variance, measurement precision, and biological heterogeneity, and therefore cannot be represented by a single global estimate. Although Superpower simulations rely on fixed-effects ANOVA rather than the hierarchical structure of the final mixed models, they still provide a realistic approximation of the study’s sensitivity under the empirical variance and correlation structure.

The MDE-based classification further clarified which effects were both statistically significant and well-powered, and which required more cautious interpretation. Effects whose CIs lay entirely outside the MDE band can be considered robust, whereas those with CIs overlapping the MDE band were classified as “significant but uncertain”, indicating that the observed differences may be smaller than the study was adequately powered to detect. Therefore, incorporating MDE thresholds helps distinguish between genuinely well-supported effects and those for which additional data would be needed to draw firm biological conclusions.

Notably, the simulated trait-specific power did not always align with MDE classification. Some trait effects with high simulated power (>0.80) and strong statistical significance (*p* < 0.01) were still placed in the “uncertain” category because their CIs overlapped the MDE band. Conversely, several effects with only moderate simulated power (0.50–0.80) and variable statistical significance (*p* < 0.05) also fell into the same uncertain category. This pattern shows that both highly powered, highly significant effects and more modestly powered effects can fail to exceed the MDE threshold. This underscores that neither *p*-values nor simulated power alone indicate whether an effect is large enough to be interpreted with biological confidence. Consequently, the MDE framework provides an essential complementary perspective by evaluating the effect magnitude relative to the study’s sensitivity.

To our knowledge, retrospective power and MDE analyses are rarely applied in small experimental studies, despite their value for distinguishing well-supported effects from those limited by sample size. Integrating these approaches strengthens the interpretation of our mixed-model results and provides a quantitative basis for designing future studies with adequate power to detect biologically meaningful effects.

A growing body of evidence indicates that neither CASA nor flow cytometry traits provide a consistent or universal predictor of fertility in poultry. Although sperm motion analysis is valuable, CASA traits alone show limited and context-dependent predictive power [39]. Empirical studies illustrate this variability. Ehling et al. reported fertilization rates of 43.6–52.1% using pooled semen, with outcomes influenced by the straw number and CPAs [31]. When similar roosters were used at an older age but inseminated into a different female group, fertilization success increased to 81.1%, despite comparable CASA profiles, highlighting the strong influence of female and age effects. In their later study of individual ejaculates, Ehling et al. found substantial line-level differences and rooster-level variation in fertility (9.7–73.3% in the lowest fertility line; 41.4 to 87.5% in the highest) [32]. Motility and progressive motility correlated with fertility in only two of three lines, and in the line with the lowest fertility, all CASA traits were correlated with fertility, whereas in the higher-fertility lines, these associations were weaker or absent. These correlations were based on breeding line averages rather than rooster-specific pairings, further complicating interpretation. More recently, Madeddu et al. reported both breed and rooster-within-breed differences in fertility and embryo viability, with overall means of 16.43% and 78.26%, respectively [34]. Together, these findings show that, while CASA traits provide useful information about sperm performance, they do not offer a reliable or universal predictor of fertilization success in poultry.

The mixed-model results showed clear rooster-specific variation in CASA traits, and the correlation analyses provided additional insight into how these traits may covary within each breed. However, given the very limited sample size, these correlation patterns should be interpreted with considerable caution. The findings are exploratory in nature and should not be regarded as definitive without further validation in larger datasets. Overall, the correlation structure differed between JH and NB8, and these differences were often treatment-dependent. MOT proved to be a poor predictor of underlying kinematic performance. In JH, PROG showed consistent positive correlations with all kinematic traits in both treatments. In contrast, NB8 displayed weak and non-significant PROG-kinematics correlations in T1, but strong and significant ones in T2, suggesting that in T2, higher PROG was more consistently associated with faster and more direct sperm movement. The correlations among the kinematic traits themselves were generally high in both breeds, but several associations shifted between treatments. In NB8, the low VCL/VAP–STR/LIN correlations in T1 became significant in T2 because VSL increased proportionally faster than VAP and VCL after refrigeration, strengthening the relationship between forward velocity and trajectory straightness. In contrast, JH showed the opposite pattern for some velocity–linearity relationships, consistent with VAP and VCL increasing more relative to VSL in T2, which weakened the STR and LIN associations. The correlations between VIA and the CASA traits were uniformly weak and non-significant in both breeds, indicating that viability is largely independent of the motility–kinematic phenotype.

The PCA revealed three statistically interpretable axes of sperm performance. PC1 represented overall movement quality, with strong positive loadings for all velocity and linearity traits, while PC2 captured a statistical contrast between motility-related and linearity-related traits. PC3 described a smaller contrast between the curvilinear velocity (VCL and VAP) and progressive movement (PROG, STR). Together, PC1–PC3 explained nearly all the phenotypic variation in CASA traits.

Treatment had a strong effect on PC1, confirming that short-term refrigerated holding reduced overall movement quality. Although the mean PC1 did not differ between breeds, the spaghetti plots revealed clear differences in within-breed variability: NB8 roosters showed shorter, more tightly clustered trajectories, whereas JH roosters displayed larger and more heterogeneous shifts. This visual pattern aligns with the high conditional R^2^ values and indicates substantial rooster-level variation in baseline quality and treatment sensitivity. For PC2, both breed and treatment had moderate to large effects, consistent with the proportionality shifts observed in the correlation analysis and visible in the PCA spaghetti plots as a consistent upward movement of NB8 roosters along PC2. The PCA biplots further supported these patterns, with treatment groups separated mainly along PC1, and breed differences were concentrated along PC2, consistent with their contrasting motility–linearity profiles.

Li et al. similarly reduced 14 fresh-semen traits from seven chicken breeds into three major factors explaining 75.8% of the variance [35]. Their Factor 1 was dominated by linearity traits, Factor 2 by velocity and head-movement traits, and Factor 3 by ejaculate quantity traits. Fertility correlated strongly with quantitative and linearity factors but only weakly with the velocity factor, suggesting unequal contributions of different sperm performance components to fertilization success. Madeddu et al. also identified distinct multivariate patterns: in fresh semen, PC1 was driven by VCL and VAP, and PC2 by sperm membrane integrity and progressive motility, with only fresh semen traits explaining breed differences [34].

To verify the mixed-model results, the PCA loadings were separately examined for each breed (Table S1). The breed-wise PCA loadings closely matched the across-breed PCA. Both breeds showed the same underlying structure, with PC1 representing overall movement quality and PC2 capturing the motility–linearity contrast. Minor differences in loading strength were present, but the organization of traits was effectively identical.

One important limitation of this study was the absence of molecular sperm-integrity assessments, such as DNA fragmentation, chromatin integrity, protamination status, and oxidative-stress markers. These parameters may be particularly informative in situations where sperm motility appears acceptable, but fertility rates are low. Flow-cytometric viability was assessed only at T1, preventing evaluation of whether short-term post-thaw refrigerated holding affected plasma membrane integrity over time. Future studies should therefore integrate CASA, flow cytometry, DNA integrity assays (e.g., SCSA or TUNEL), oxidative-stress markers, viability assessments at all post-thaw time points, and fertility outcomes to clarify the biological basis of reduced fertilizing capacity in cryopreserved rooster semen.

## 5. Implications

Our study revealed nuanced differences in the interdependency of the motility–velocity–kinematic complex between breeds. Both mixed models and PCA confirmed strong between-rooster variation. Additionally, short-term refrigerated holding significantly affected many of these traits and the coherence of motility and kinematic traits, particularly in NB8. Generally, the interpretation of a small-scale pilot study using a comprehensive set of statistical methods benefited from a detailed examination of statistical power and trait-specific MDEs. These results have important implications for decisions regarding experimental design, the choice of statistical methods, and the development of cryobanking strategies.

The breed differences in covariation, strong effects of short-term post-thaw refrigerated holding, and strong between-rooster variation observed here highlight the need for careful experimental design in future studies on rooster sperm quality for gene banking. Future studies should incorporate a sufficient number of males per breed to ensure adequate statistical power. Given the multivariate nature of sperm performance, reliance on single traits may obscure biologically relevant patterns; instead, multivariate approaches such as PCA, factor analysis, or clustering should be integrated into routine analyses. Our results also emphasize the importance of repeated sampling, because any trait—or composite quality index—that might eventually be used as a predictor of fertilization success must show consistent individual coherence across minor protocol variations. If short-term refrigerated holding alters this coherence, as observed here, estimates of individual performance could be biased and the predictive value of such indices weakened.

These findings also carry direct relevance for cryoconservation strategies for endangered livestock breeds. Because each rooster contributes a distinct multivariate profile of sperm performance, cryobanks that rely on only a few donors risk capturing a narrow subset of the breed’s reproductive phenotype. This may inadvertently reduce the functional diversity available for future breed recovery or assisted reproduction. Our results therefore reinforce the principle in the FAO cryoconservation manual that gene banks should sample broadly across many donors to maximize genetic representativeness, rather than accumulating large numbers of doses from a small number of individuals [40].

From a population-level perspective, maintaining an adequate number of donors is essential to mitigating risks associated with genetic load, inbreeding depression, and demographic or environmental stochasticity [41]. At present, no supplementary cryopreservation efforts are undertaken in the Norwegian live gene bank for laying hens, largely due to the very poor fertilization success of earlier trials. To develop effective long-term cryoconservation practices, a necessary next step is to conduct a larger, integrated study that combines our current findings on sperm trait variability with comprehensive assessments of both fresh and frozen–thawed semen quality. Crucially, such a study should be paired with a fertilization trial to directly link semen characteristics with reproductive outcomes. This approach would provide the empirical foundation needed to refine donor selection, improve cryopreservation protocols, and ultimately enable more reliable conservation of reproductive potential in endangered poultry breeds.

## 6. Conclusions

This pilot study shows that objective semen-quality assessments using CASA and flow cytometry (LIVE/DEAD) can detect both line-associated differences and post-thaw handling effects in cryopreserved rooster semen from conserved Norwegian lines. Short-term post-thaw refrigerated holding reduced several motility and velocity traits, and multivariate analyses revealed major dimensions of covariance among CASA-derived sperm-quality parameters. These findings support the value of objective quality-control tools in poultry cryobanking, although they do not explain the biological causes of previously observed low fertility. Future work with larger sample sizes should integrate repeated sampling, fertility trials, molecular markers of sperm integrity, and information on genetic relatedness to clarify the reproductive relevance of the semen-quality patterns identified here.

## Figures and Tables

**Table 1:** Descriptive statistics and mixed-model results for sperm motility, and kinematic and viability traits in Jærhøns (JH) and NorBrid8 (NB8) roosters under two post-thaw holding treatments (T1 and T2).

Trait	JH		NB8			*p*-Value	
	T1	T2	T1	T2	Overall	Breed	Treatment
MOT (%)							
Mean	43.52	37.25	36.07	29.00	36.46	0.011 *	0.000 ***
SD	7.80	6.86	7.92	7.11	8.88		
CV (%)	17.92	18.41	21.96	24.53	24.36		
PROG (%)							
Mean	24.59	20.40	19.21	15.57	19.94	0.017 *	0.000 ***
SD	6.72	5.30	3.47	4.39	5.92		
CV (%)	27.32	25.98	18.06	28.17	29.68		
VCL (µm/s)							
Mean	66.41	61.69	64.50	61.67	63.57	0.677	0.007 **
SD	7.99	6.07	5.95	5.48	6.56		
CV (%)	12.04	9.84	9.22	8.89	10.31		
VAP (µm/s)							
Mean	50.36	44.88	48.92	45.86	47.50	0.929	0.005 **
SD	9.02	6.84	6.17	6.28.	7.29		
CV (%)	17.90	15.24	12.61	13.69	15.35		
VSL (µm/s)							
Mean	36.40	31.54	36.54	33.21	34.42	0.734	0.002 **
SD	9.64	6.22	5.94	5.99	7.22		
CV (%)	26.48	19.74	16.27	18.04	20.97		
STR (%)							
Mean	68.02	67.11	67.56	66.37	67.27	0.762	0.240
SD	5.46	6.02	4.99	4.49	5.13		
CV (%)	8.02	8.96	7.38	6.76	7.63		
LIN (%)							
Mean	50.94	48.75	51.15	49.80	50.16	0.780	0.040 *
SD	6.72	6.14	5.16	5.28	5.75		
CV (%)	13.19	12.59	10.08	10.61	11.47		
VIA (%)							
Mean	65.64	NA	60.21	NA	62.93	0.149	NA
SD	8.88	NA	8.92	NA	9.14		
CV (%)	13.53	NA	14.82	NA	14.52		

MOT, total motility; PROG, progressive motility; VCL, curvilinear velocity; VAP, average path velocity; VSL, straight-line velocity; STR, straightness; LIN, linearity; VIA, viability; *, **, *** indicate statistical significance at *p* < 0.05, *p* < 0.01, and *p* < 0.001, respectively.

**Table 2:** Principal component analysis (PCA) loadings for CASA traits on the first three principal components and the variation explained by the principal component (PC). Loadings ≥ |0.35|are in bold.

Trait	PC1	PC2	PC3
MOT	0.20	**−0.73**	−0.18
PROG	**0.36**	**−0.41**	**−0.42**
VCL	**0.39**	−0.13	**0.54**
VAP	**0.43**	0.01	**0.40**
VSL	**0.45**	0.16	0.19
STR	**0.36**	**0.35**	**−0.49**
LIN	**0.40**	**0.36**	−0.26
Variance explained (%)	65.69	20.80	11.79

**Table 3:** Mixed-model *p*-values and partial effect sizes (η^2^_p_) for breed and treatment effects on PCA scores.

	*p*-Value		η^2^_p_		R^2^ (%)	
PC	Breed	Treatment	Breed	Treatment	Marginal	Conditional
PC1	0.532	<0.001 ***	0.02	0.44	10.48	76.40
PC2	0.018 *	0.001 **	0.23	0.37	24.40	77.92
PC3	0.120	0.790	0.11	0.00	6.88	35.63

*, **, *** indicate statistical significance at *p* < 0.05, *p* < 0.01, and *p* < 0.001, respectively.

## Data Availability

The analyses in this study are based on original data collected for this project. All summary statistics reported in the manuscript accurately reflect the underlying dataset. Additional information or further data details can be provided by the authors upon reasonable request.

## Supplementary Materials

The following supporting information can be downloaded at: https://www.mdpi.com/article/10.3390/ani16142181/s1. Table S1: Principal component analysis (PCA) loadings for CASA traits for Jærhøns and NorBrid8 on the first three principal components and the variation explained by the principal component (PC). Loadings ≥ |0.35| are on bold. Figure S1A–H: Observed effect estimates (±95% CI) for the breed (A) and treatment (B) effects compared with the minimum detectable effect (MDE) thresholds for CASA and flow cytometry traits. Dashed lines indicate positive and negative MDE values, with bold labels showing their magnitudes. These MDE values are derived from the analytical power calculations. Numerical point estimates and significance indicators are shown above each effect; *, **, *** indicate statistical significance at *p* < 0.05, *p* < 0.01, and *p* < 0.001, respectively. MOT: total motility (%), PROG: progressive motility (%), VCL: curvilinear velocity (μm/s), VAP: average path velocity (μm/s), VSL: straight line velocity (μm/s), STR: straightness ((VSL/VAP)*100, %), LIN: linearity ((VSL/VCL)*100, %), and VIA: viability (%). Figure S2: Pairwise correlation coefficients for CASA-derived traits at T1 and T2. Correlations for Jærhøns (JH) are shown in red, and those for NorBrid8 (NB8) in blue; *, **, *** indicate statistical significance at *p* < 0.05, *p* < 0.01, and *p* < 0.001, respectively.

## Abbreviations

The following abbreviations are used in this manuscript:

ANOVA	Analysis of variance
CASA	Computer-assisted sperm analysis
CI	Confidence interval
CPAs	Cryoprotectant agents
DMA	Dimethylacetamide
EEA	European Economic Area
FAO	Food and Agriculture Organization of the United Nations
FCM	Flow cytometry
JH	Jærhøns, native Norwegian layer breed
MDE	Minimum detectable effect
MHC	Major histocompatibility complex
MOT	Total motility
NB8	NorBrid8 breeding line
LIN	Linearity, calculated as VSL/VCL
NIBIO	Norwegian Institute of Bioeconomy Research
PC	Principal component
PCA	Principal component analysis
PROG	Progressive motility
STR	Straightness, calculated as VSL/VAP
T1	Treatment one, CASA analyses directly after thawing
T2	Treatment two, CASA analyses ≤ 5 min refrigerated holding
VAP	Average path velocity
VCL	Curvilinear velocity
VIA	Viability
VSL	Straight-line velocity
