# Supplementary material for: Comprehensive Statistical Assessment of Post-Thaw Semen Quality in Two Conserved Norwegian Rooster Lines
Source: Animals (Basel). 2026 Jul 14;16(14):2181. doi: 10.3390/ani16142181 (PMC13405710; doi:10.3390/ani16142181)
Supplement: Supplementary file 1 [file animals-16-02181-s001.zip › animals-4386358-Supplementary materials.pdf]

Table S1: Principal component analysis (PCA) loadings for CASA traits for Jærhøns and NorBrid8 on the first three principal components and the variation explained by the principal component (PC). Loadings ≥ | 0.35 | are on bold.

Jærhøns			
Trait	PC1	PC2	PC3
MOT	0.24	**-0.72**	**-0.35**
PROG	**0.39**	-0.22	**-0.46**
VCL	**0.38**	-0.22	**0.52**
VAP	**0.41**	-0.07	**0.42**
VSL	**0.44**	0.12	0.21
STR	**0.35**	**0.47**	**-0.38**
LIN	**0.40**	**0.38**	-0.16
Variance explained (%)	69.98	16.68	11.92
Norbrid8			
Trait	PC1	PC2	PC3
MOT	-0.16	**0.70**	0.23
PROG	-0.35	**0.43**	**0.45**
VCL	**-0.40**	0.16	**-0.55**
VAP	**-0.45**	0.02	**-0.36**
VSL	**-0.46**	-0.11	-0.19
STR	**-0.36**	**-0.40**	**0.43**
LIN	**-0.39**	**-0.36**	0.31
Variance explained (%)	63.81	24.55	10.02

A. MOT

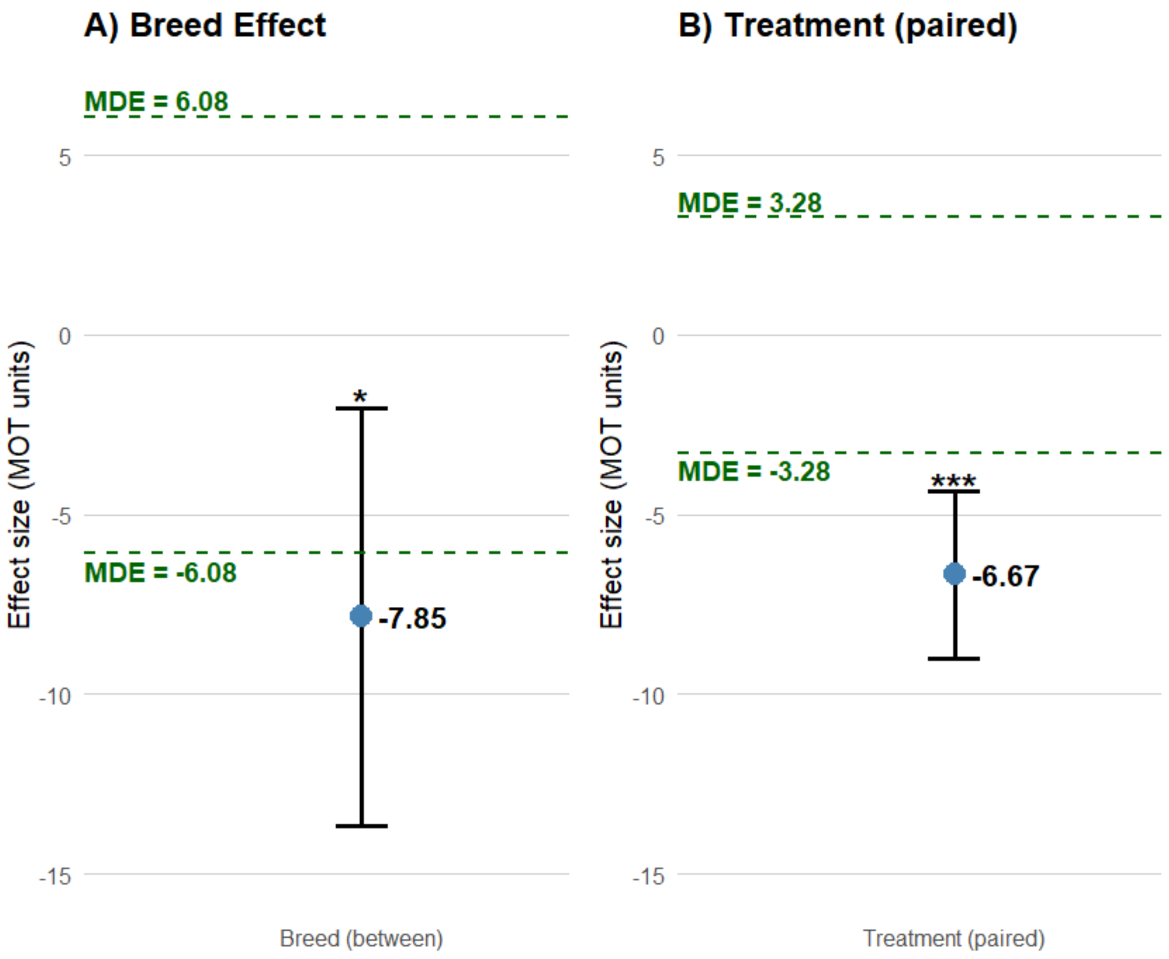

A) Breed Effect
MDE = 6.08
5
0
-5
-10
-15
Effect size (MOT units)
*
MDE = -6.08
-7.85
Breed (between)
B) Treatment (paired)
MDE = 3.28
MDE = -3.28
***
-6.67
Treatment (paired)

B. PROG

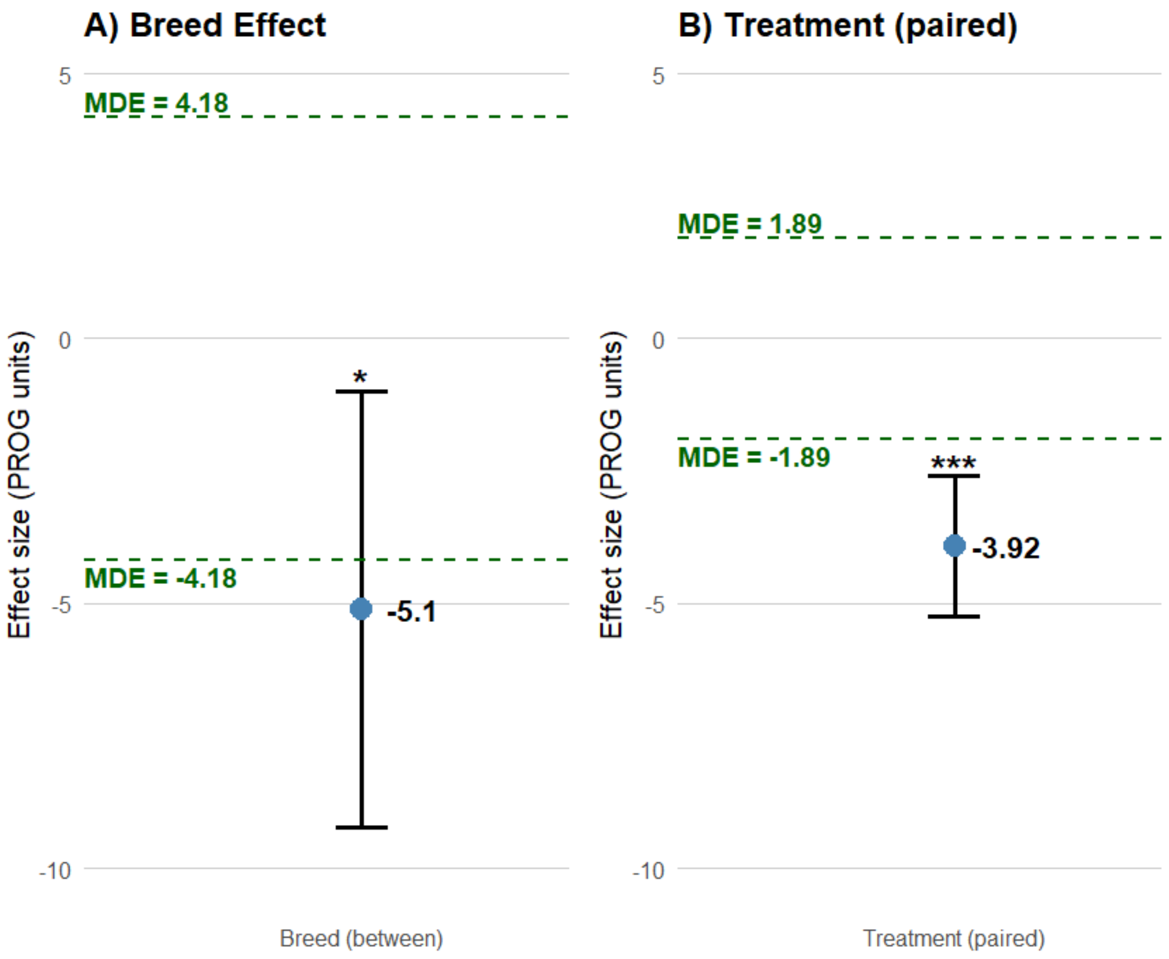
A) Breed Effect
B) Treatment (paired)
MDE = 4.18
MDE = -4.18
MDE = 1.89
MDE = -1.89
*
***
-5.1
-3.92
5
0
-5
-10
Effect size (PROG units)
Breed (between)
Treatment (paired)

C. VCL

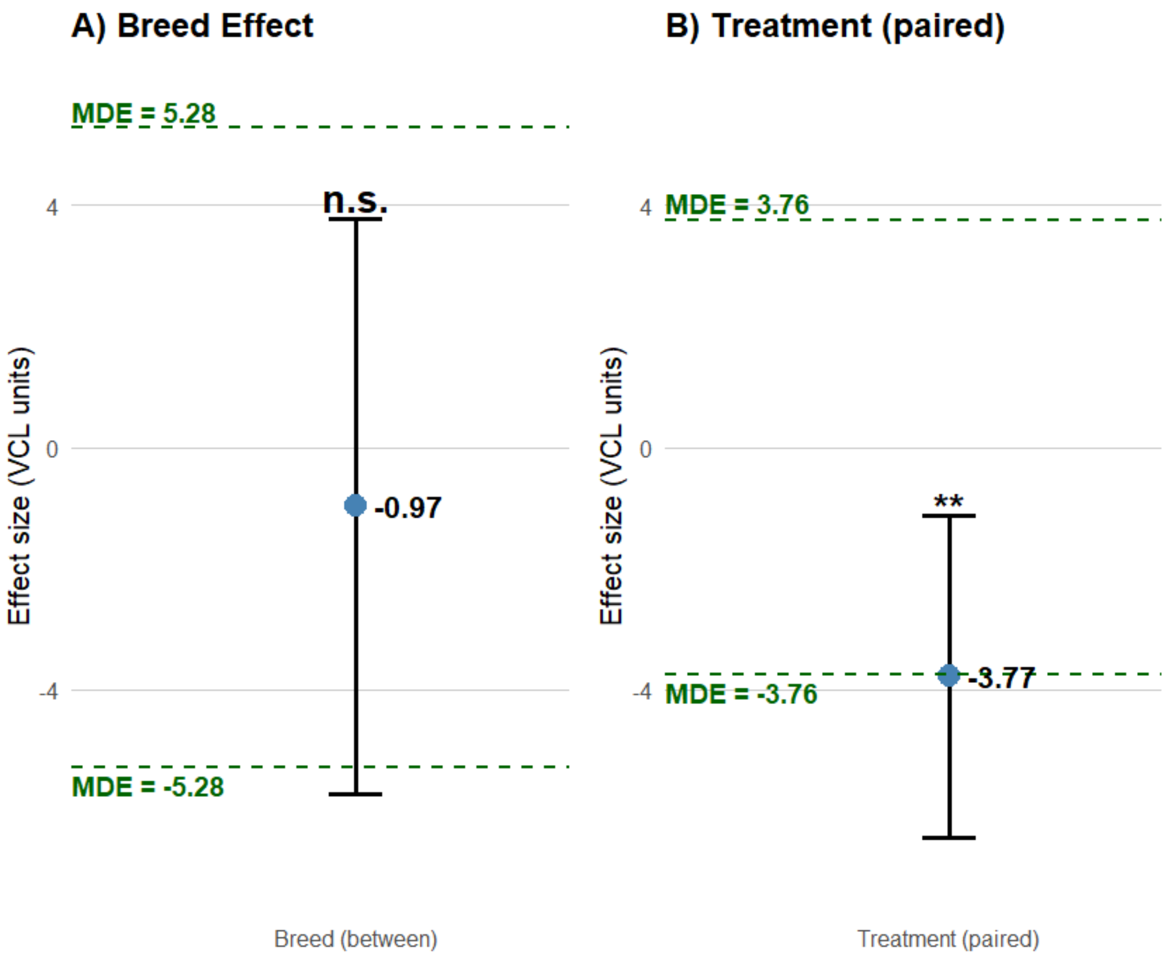
A) Breed Effect
MDE = 5.28
n.s.
-0.97
MDE = -5.28
Effect size (VCL units)
4
0
-4
Breed (between)
B) Treatment (paired)
MDE = 3.76
**
-3.77
MDE = -3.76
Effect size (VCL units)
4
0
-4
Treatment (paired)

D. VAP

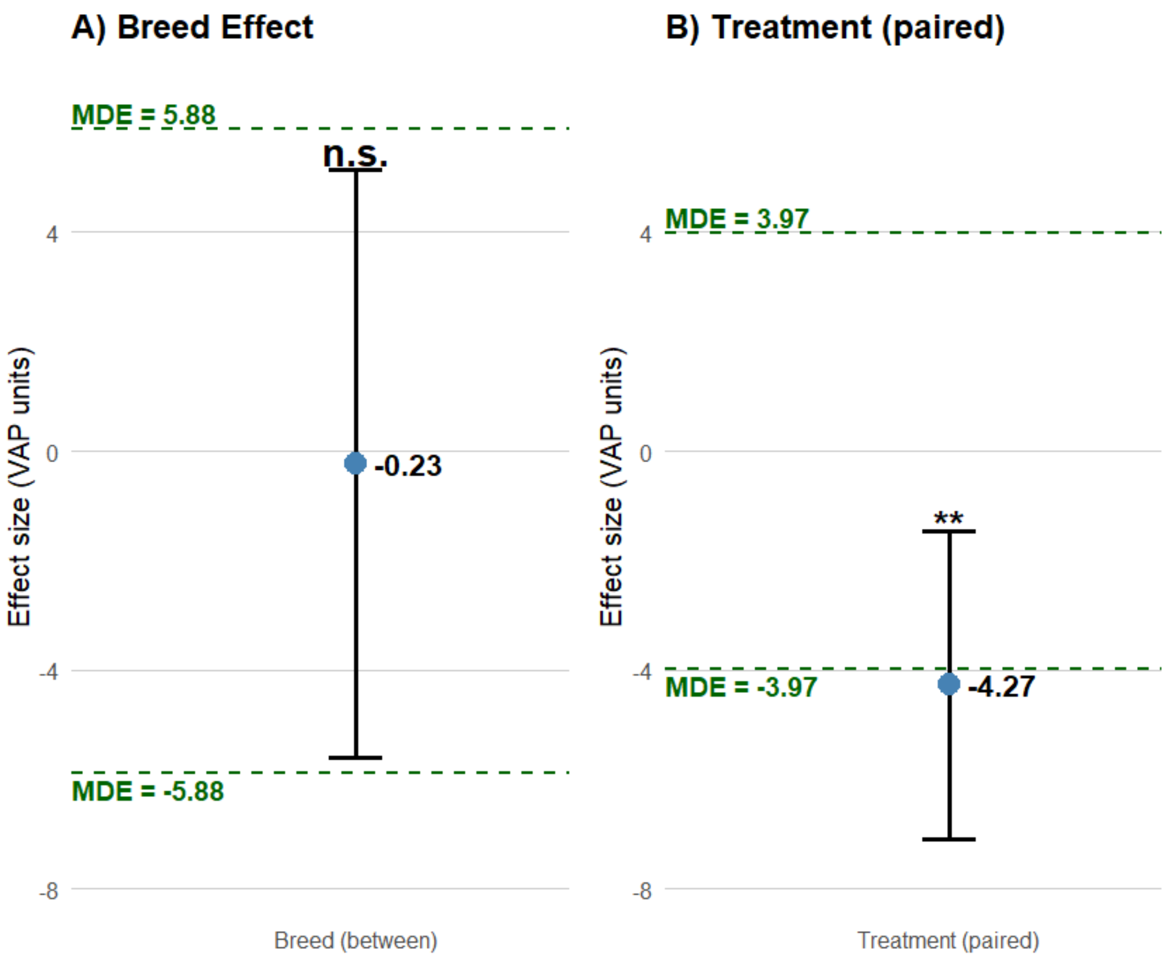

A) Breed Effect
B) Treatment (paired)
MDE = 5.88
n.s.
-0.23
MDE = -5.88
Effect size (VAP units)
4
0
-4
-8
Breed (between)
MDE = 3.97
**
MDE = -3.97
-4.27
Treatment (paired)

E. VSL

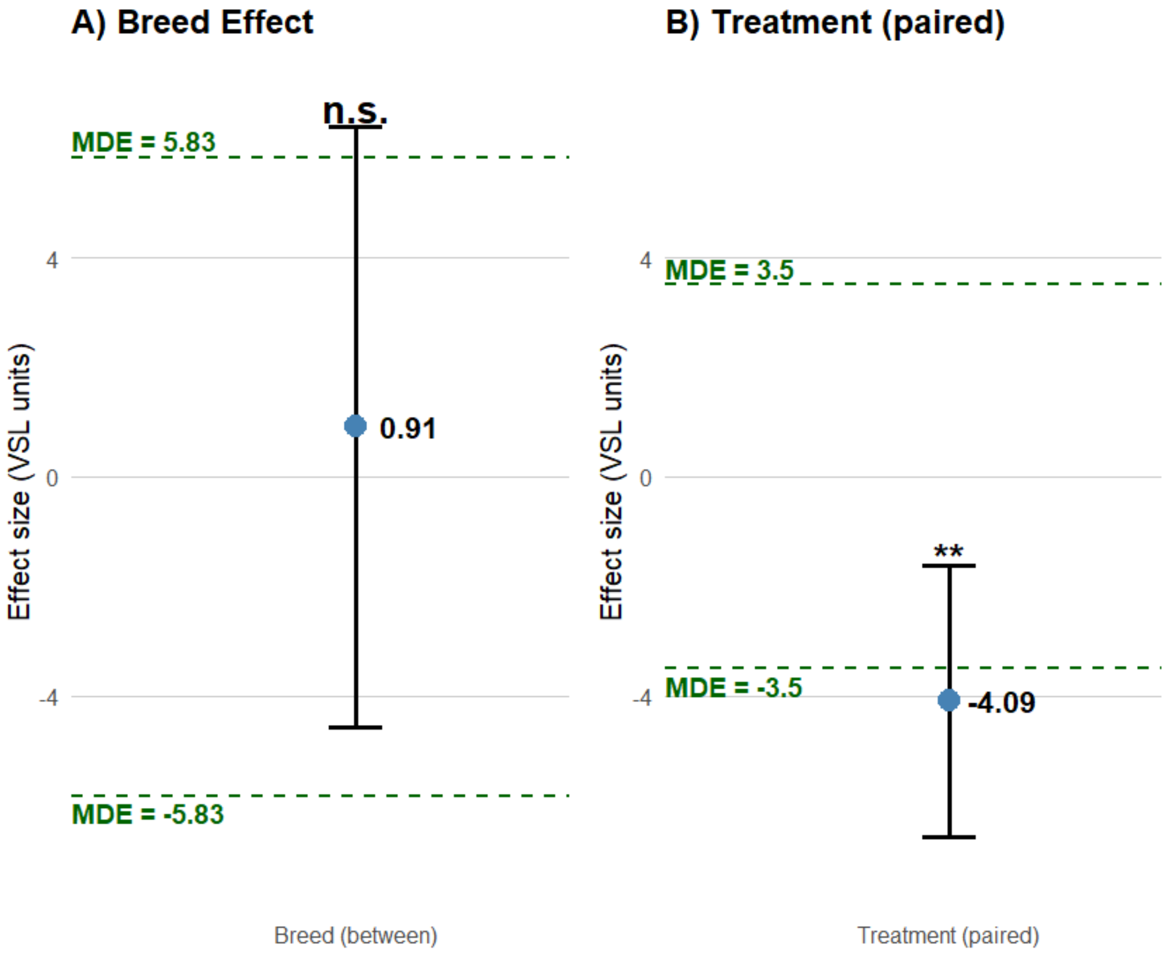
A) Breed Effect
B) Treatment (paired)
n.s.
MDE = 5.83
MDE = -5.83
0.91
Effect size (VSL units)
4
0
-4
Breed (between)
MDE = 3.5
MDE = -3.5
**
-4.09
Effect size (VSL units)
4
0
-4
Treatment (paired)

F. STR

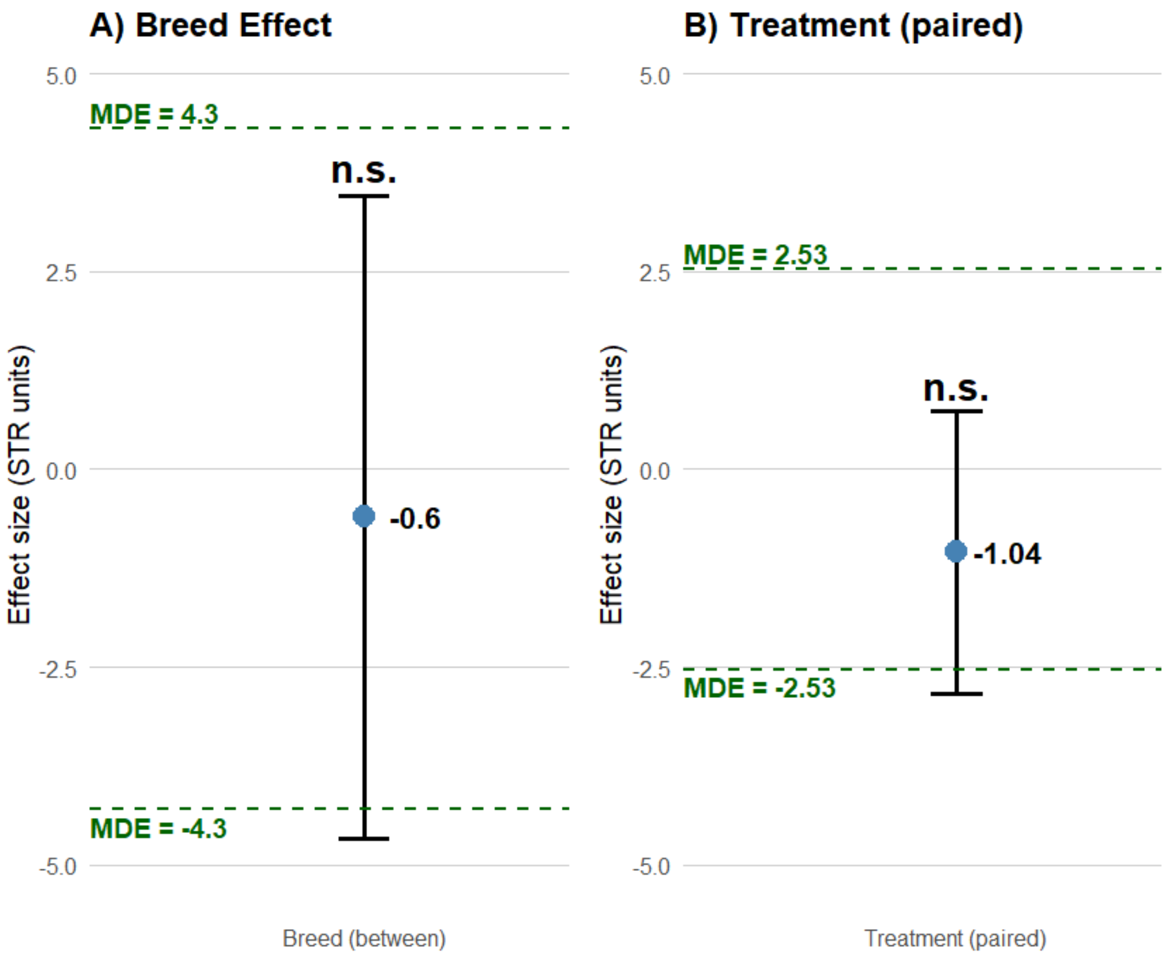
A) Breed Effect
B) Treatment (paired)
5.0
2.5
0.0
-2.5
-5.0
Effect size (STR units)
MDE = 4.3
MDE = -4.3
n.s.
-0.6
MDE = 2.53
MDE = -2.53
n.s.
-1.04
Breed (between)
Treatment (paired)

G. LIN

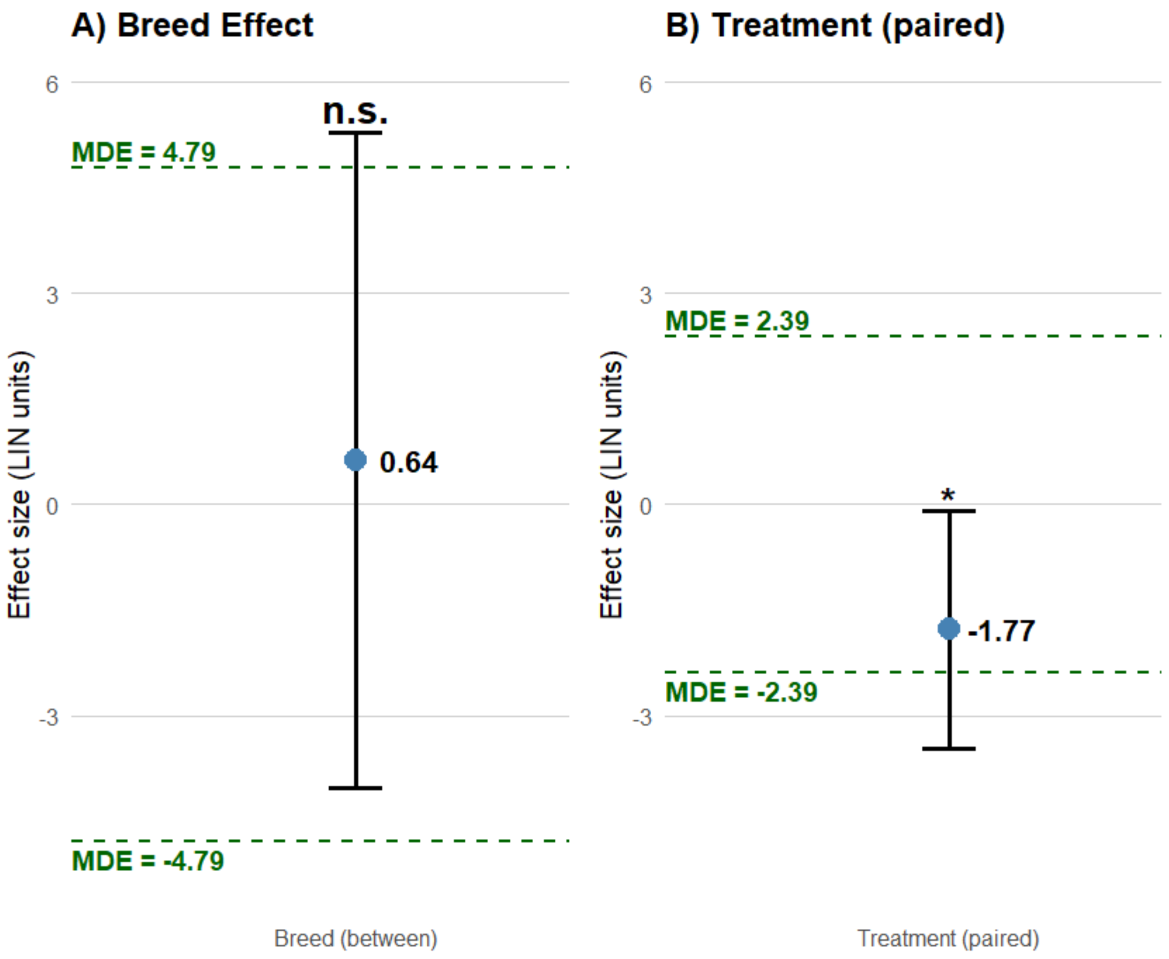
A) Breed Effect
B) Treatment (paired)
6
3
0
-3
Effect size (LIN units)
n.s.
MDE = 4.79
0.64
MDE = -4.79
Breed (between)
MDE = 2.39
*
-1.77
MDE = -2.39
Treatment (paired)

H. VIA

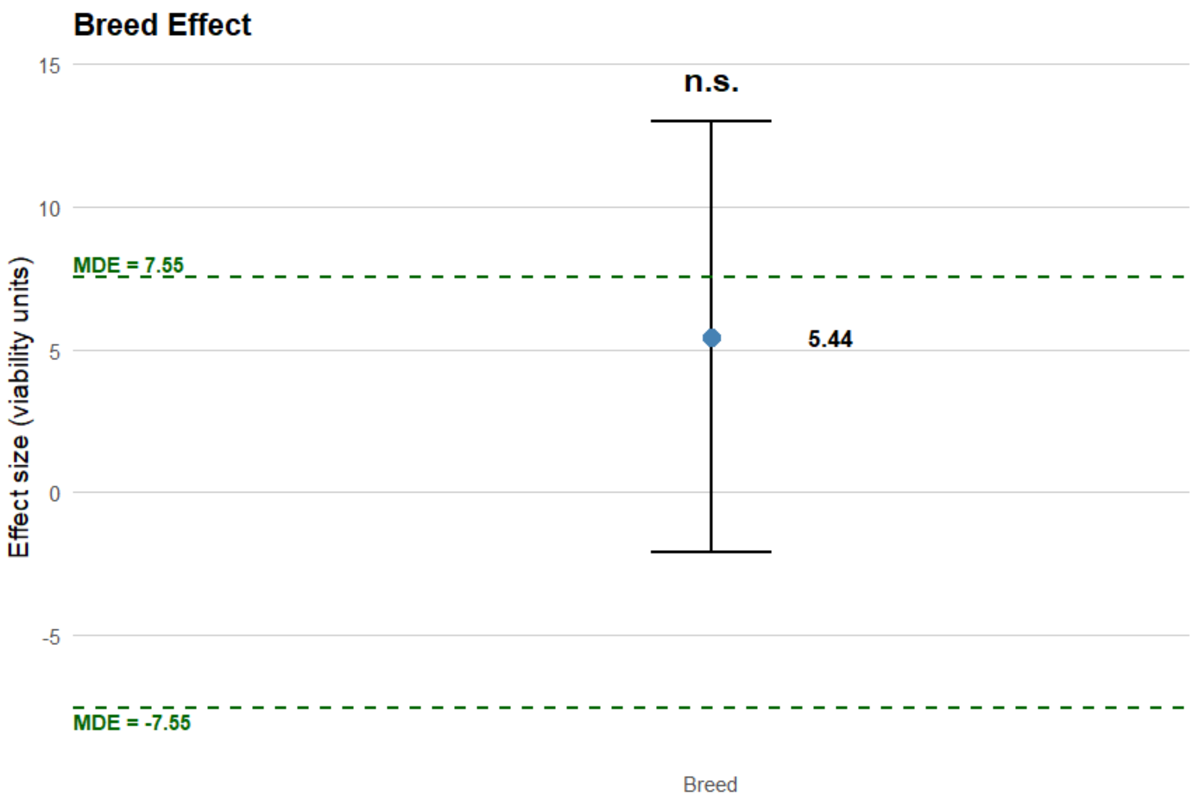

Figure S1 A-H: Observed effect estimates (±95% CI) for the breed (A) and treatment (B) effects compared with the minimum detectable effect (MDE) thresholds for CASA and flow cytometry traits. Dashed lines indicate positive and negative MDE values, with bold labels showing their magnitudes. These MDE values are derived from the analytical power calculations. Numerical point estimates and significance indicators are shown above each effect; *, **, *** indicate statistical significance at $p<0.05$, $p<0.01$, and $p<0.001$, respectively. MOT: total motility (%), PROG: progressive motility (%), VCL: curvilinear velocity (µm/s), VAP: average path velocity (µm/s), VSL: straight line velocity (µm/s), STR: straightness ((VSL/VAP)*100, %), LIN: linearity ((VSL/VCL)*100, %), and VIA: viability (%).

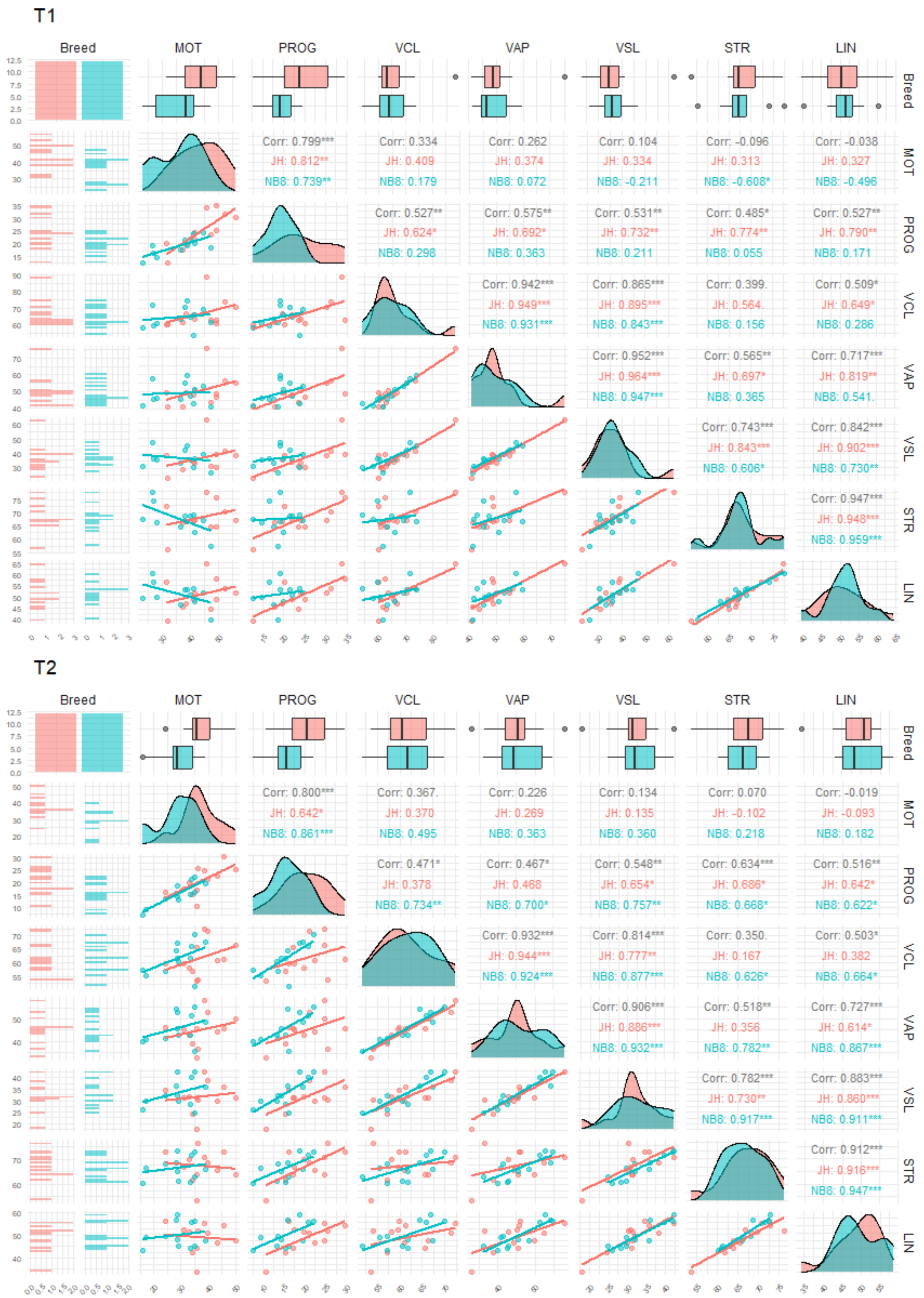

Figure S2: Pairwise correlation coefficients for CASA-derived traits at T1 and T2. Correlations for Jærhøns (JH) are shown in red, and those for NorBrid8 (NB8) in blue; *, **, *** indicate statistical significance at $p<0.05$, $p<0.01$, and $p<0.001$, respectively.
